# Power doppler ultrasound signal predicts abnormal HDL function in patients with rheumatoid arthritis

**DOI:** 10.1007/s00296-023-05285-7

**Published:** 2023-02-24

**Authors:** Christina Charles-Schoeman, Jennifer Wang, Ani Shahbazian, Holly Wilhalme, Jenny Brook, Gurjit S. Kaeley, Buzand Oganesian, Ami Ben-Artzi, David A. Elashoff, Veena K. Ranganath

**Affiliations:** 1 David Geffen School of Medicine, University of California, 1000 Veteran Ave, Rm 32-59, Los Angeles, CA 90095 USA; 2 Division of Rheumatology and Clinical Immunology, College of Medicine, University of Florida, Jacksonville, FL USA; 3 Division of Rheumatology, Cedars Sinai Medical Center, Los Angeles, CA USA

**Keywords:** Rheumatoid arthritis, Ultrasound, High-density lipoprotein

## Abstract

Active rheumatoid arthritis (RA) is associated with increased cardiovascular risk and impaired function of high-density lipoprotein (HDL). Previous work suggests that HDL may become dysfunctional through oxidative modifications within the RA joint. The current work evaluates an association of synovial power doppler ultrasound signal (PDUS) with HDL function and structure. Two open-label clinical therapeutic studies using PDUS as a disease outcome measure were included in this analysis, including a 12-month trial of subcutaneous abatacept in 24 RA patients and a 6-month trial of IV tocilizumab in 46 RA patients. Laboratory assays included assessments of HDL function and structure, HDL and total cholesterol levels, and a cytokine/chemokine panel. Patients with the highest baseline PDUS scores in both clinical studies, had worse HDL function, including suppression of paraoxonase 1 (PON1) activity as well as lower HDL-C levels. Associations between other disease assessments (DAS28 and CDAI) and HDL function/structure were noted but were generally of lesser magnitude and consistency than PDUS across the HDL profile. Treatment with tocilizumab for 6 months was associated with increases in cholesterol levels and improvements in the HDL function profile, which correlated with greater decreases in PDUS scores. Similar trends were noted following treatment with abatacept for 3 months. Higher baseline PDUS scores identified patients with worse HDL function. This data supports previous work suggesting a direct association of joint inflammation with abnormal HDL function.

**Supplementary Information:**

The online version contains supplementary material available at 10.1007/s00296-023-05285-7.

## Introduction

Patients with rheumatoid arthritis (RA) have an increased risk of cardiovascular disease (CVD) and accelerated atherosclerosis compared to the general population [1–3]. High RA disease activity over time is associated with increases in CVD [4–6], however, a better understanding of the specific mechanisms, which drive this association is greatly needed. Traditional cholesterol levels may be suppressed in the setting of active RA, further complicating the clinical CV risk assessment.

High-density lipoprotein (HDL) is an anti-inflammatory particle, which protects against atherosclerosis and regulates systemic inflammation through multiple mechanisms [7–10]. HDL contains multiple associated proteins, which normally perform its protective roles, including the enzyme paraoxonase 1 (PON1), which protects against the oxidation of lipoproteins and neutralizes oxidized species [11–16]. However, in the setting of active RA, the function of HDL becomes abnormal, and impaired PON1 activity in RA patients is associated with increased cardiovascular risk [12, 17, 18].

In the current work, we hypothesized that HDL may become abnormal in the joint itself through modifications caused by the inflammatory, pro-oxidant synovial environment in patients with active RA. We used musculoskeletal ultrasound (MSUS) to quantify active synovitis through Power Doppler (PDUS) in RA patients treated in two clinical therapeutic trials to assess the relationship between active synovitis and abnormal HDL function and structure.

## Materials and methods

### Study design

All subjects gave written informed consent for the study under protocols approved by the Human Research Subject Protection Committee at UCLA.

#### Abatacept study

PDUS assessments were performed on 24 RA patients naive to biologics in a 12-month, single-center, open-label study of subcutaneous abatacept as previously described [19]. All patients were started at baseline on abatacept 125 mg SQ week dosing. Low-dose prednisone (≤ 10 mg daily) was allowed during the study but was required to remain at a stable dose during the study duration. Seven joints were scanned on the most affected side (wrist, MCP joint 2/3, PIP joint 2/3 and MTP joint 2/5) according to Backhaus et al. (PDUS-7, range 0–21) [20] using a GE LogicE9 machine (M6-15 MHz linear probe, General Electric Healthcare, Chicago, IL). Standardized settings were used for each visit for all patients. PDUS was scored semiquantitatively according to published consensus definitions. The sonographer (AB) was blinded to the clinical data, and the clinical assessor was blinded to PDUS-7 scores. The weighted Kappa intra-reader reliability was 0.82.

#### Tocilizumab study

PDUS also was assessed in 46 RA patients in an open-label, 2-site, 6-month study of intravenous (IV) tocilizumab. Approximately 82.6% were biologic DMARD or Janus kinase inhibitors experienced (Supplementary Table 1). Low-dose prednisone (≤ 10 mg daily) was allowed during the study but was required to remain at a stable dose during the study duration. At baseline, patients were started on IV tocilizumab 4 mg/kg and were dose escalated to 8 mg/kg if at 12 weeks the DAS28 > 3.2. Only 3 patients were not dose escalated at 12 weeks. Thirty-four joints were scanned (bilateral wrists, radioulnar, MCP1-5, IP, PIP2-5, knees, and MTP2-5) in a similar semiquantitative manner using MyLab70C machine (12–18 MHz linear probe) using the LAJAX (pronounced ‘Lay-Jax’) 34 joint protocol (PDUS-34, range 0–102) [21]. The same ultrasound machine and standardized settings were used at both sites and the sonographers were blinded to clinical assessments (AB, VKR, GK). Similarly, the sonographers and the clinical assessors were blinded to one another. The weighted Kappa inter-reader reliability was 0.77 and the intra-reader reliability ranged between 0.82 and 0.89 for the PDUS assessments.

#### Biorepository

Blood was collected in heparinized tubes (Becton Dickinson) at baseline and the end study visits and stored at − 80 °C for the assays described below.

### Clinical data

Patient demographic characteristics were obtained at the screening visit. All patients had the following assessments at baseline and end of study visits: 28 tender joint count (TJC 28), 28 swollen joint count (SJC 28), patient global visual analogue scale (VAS), erythrocyte sedimentation rate (ESR), and physician global VAS. The four variable disease activity scale using 28 joint count and ESR (DAS28/ESR-4 item) and clinical disease activity index (CDAI) were also calculated.

### Evaluation of HDL’s anti-oxidant function

The cell-free assay was a modification of a previously published method using LDL as the fluorescence-inducing agent [22]. HDL was isolated by dextran bead precipitation. To determine the anti-inflammatory properties of HDL, the change in fluorescence intensity as a result of the oxidation of 2′,7′-dichlorodihydrofluorescein diacetate (H2DCFDA) (ThermoFisher Scientific) to 2′,7′-dichlorofluorescein (DCF) in incubations with a standard LDL in the absence or presence of the test HDL was assessed and the HDL inflammatory index (HII) calculated. Readings with H2DCFDA and LDL cholesterol were normalized to 1.0. In brief, as described previously, 25 μl of LDL-cholesterol (100 μg/ml) was mixed with 50 μl of test HDL (100 μg HDL-cholesterol/ml) in black, flat bottom polystyrene microtitre plates and incubated at 37 °C with rotation for 30 min. Twenty-five microlitres of H2DCFDA solution (0.2 mg/ml) was added to each well, mixed, and incubated at 37 °C for 1 h with rotation. Fluorescence was determined with a plate reader (Spectra Max, Gemini XS Molecular Devices) at an excitation wavelength of 485 nm, emission wavelength of 530 nm and cutoff of 515 nm with photomultiplier sensitivity set at medium. Values for intra- and interassay variability were 0.5 (0.37)% and 3.0 (1.7)%, respectively [23].

### Determination of PON1 activity

PON1 activity was quantified using three substrates (paraoxon, dihydrocoumarin and phenylacetate) to assess its paraoxonase, lactonase and arylesterase activities, respectively, as described previously [24].

### HDL-associated apolipoprotein AI (HDL-ApoAI) and HDL associated Haptoglobin (HDL-Hp)

HDL-ApoAI and HDL-Hp were measured by sandwich ELISA as described previously with minor modifications [25]. All antibodies were purchased from Genway Biotech.

### Cholesterol and cytokine/chemokine assays

Total and HDL cholesterol were calculated using standard colorimetric assays [26, 27]. Plasma cytokine and chemokine levels including IFN-gamma, IL-10, MIP3a, IL12p70, IL-13, IL-15, IL-17a, IL-6, IL-17E, IL-27, IL-31, TNF-a, and IL-28a were measured using a luminex multiplex assay (Fisher Scientific).

### Statistical analysis

Data were analyzed using SAS Version 9.4 (SAS Institute Inc., Cary, NC, USA). Groups were compared using Analysis of variance (ANOVA) for continuous variables and the Chi-square test for categorical variables and Fisher’s exact test for small sample sizes. When needed, nonparametric Kruskall-Wallistests were used to analyze continuous variables. Correlations between variables were evaluated using Spearman’s correlation coefficient. Paired *t* tests were used to compare baseline to follow-up measurements. The significance level was pre-specified at *p* < 0.05.

## Results

### Clinical characteristics of abatacept and tocilizumab cohorts

Overall patient characteristics of the abatacept and tocilizumab cohorts were similar across age, gender, race/ethnicity, and seropositivity (all *p* ≥ 0.05) (Supplementary Table 1). The abatacept trial did not include patients who had received prior biologics and patients’ RA disease duration was shorter (*p* < 0.001). The patients enrolled in the tocilizumab trial had significantly higher BMI compared to patients in the abatacept trial (BMI 25.7 vs 30.3 kg/m^2^).

### Higher PDUS scores associate with impaired HDL function and decreased PON1 activity

#### Abatacept study

**Fig. 1:**
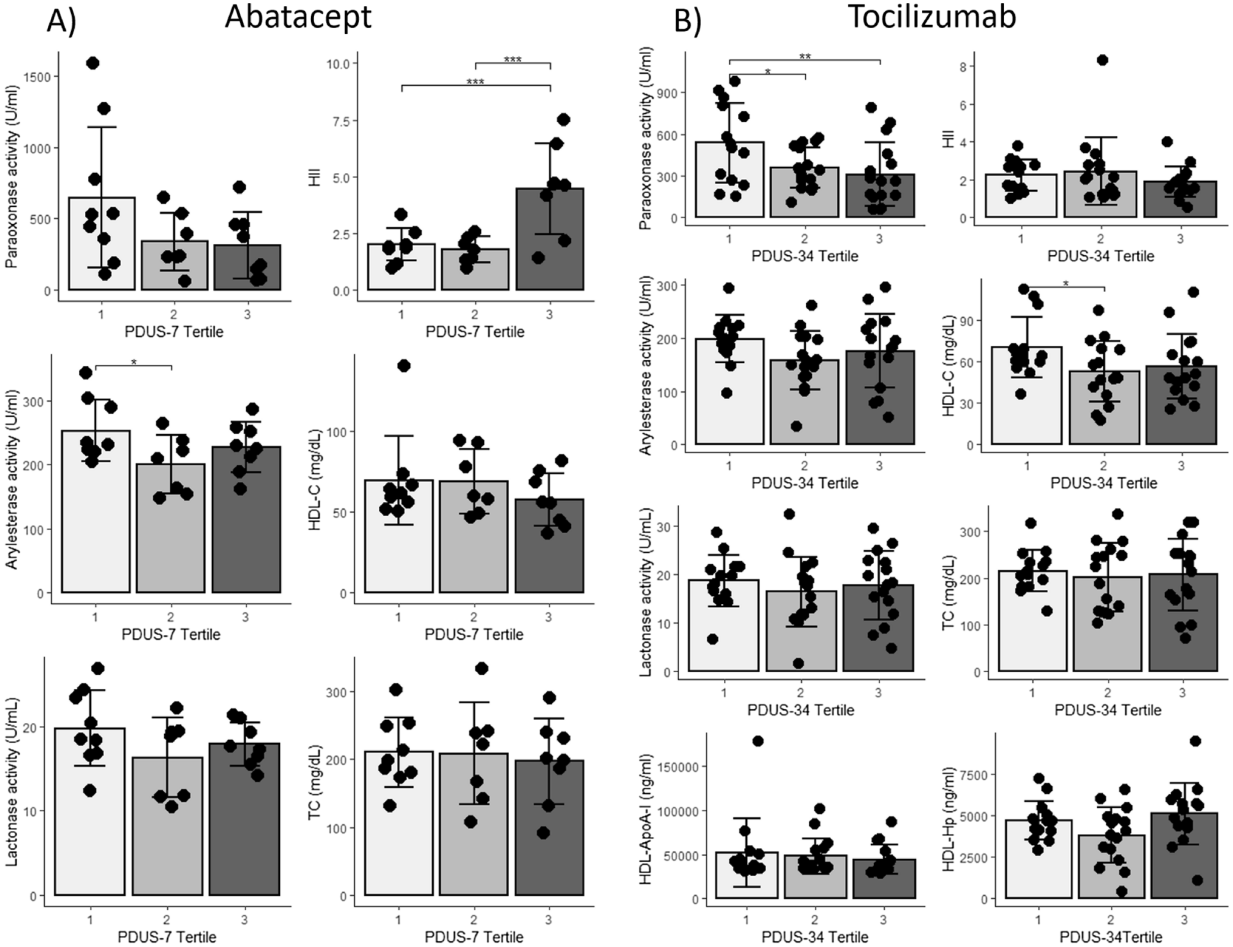
Measures of HDL function, PON1 activity and cholesterol grouped by PDUS tertiles. Panel **A **shows paraoxonase, arylesterase, and lactonase activities, HII, HDL-C, and TC in patients participating in the abatacept study grouped by PDUS-7 tertile. Panel **B** shows paraoxonase, arylesterase, and lactonase activities, HII, HDL-C, TC, HDL-ApoA-I, and HDL-Hp ﻿in patients participating in the tocilizumab study grouped by PDUS-34 tertile. *p < 0.05 **p < 0.01 ***p < 0.001; pairwise comparisons conducted when ANOVA is statistically significant

Patients with the highest baseline PDUS-7 scores (third tertile PDUS) had significantly worse HDL function as measured by a higher HDL inflammatory index (HII) compared to patients with lower baseline PDUS-7 signal in the first and second PDUS tertiles (Fig. 1A). Higher PDUS-7 scores were significantly correlated with a higher HII (*r* = 0.50, *p* = 0.01). The activity of PON1, a major anti-oxidant protein of HDL, was suppressed in patients with high PDUS-7 scores (Fig. 1A), and a significant inverse correlation was noted between PDUS-7 scores and PON1 activity (r = − 0.45, *p* = 0.03); higher PDUS-7 scores were associated with lower PON1 activity measured by the paraoxonase assay. Clinical characteristics including demographics, RA disease characteristics, and medication use were similar between PDUS tertile groups (Supplementary Table 2).

**Fig. 2:**
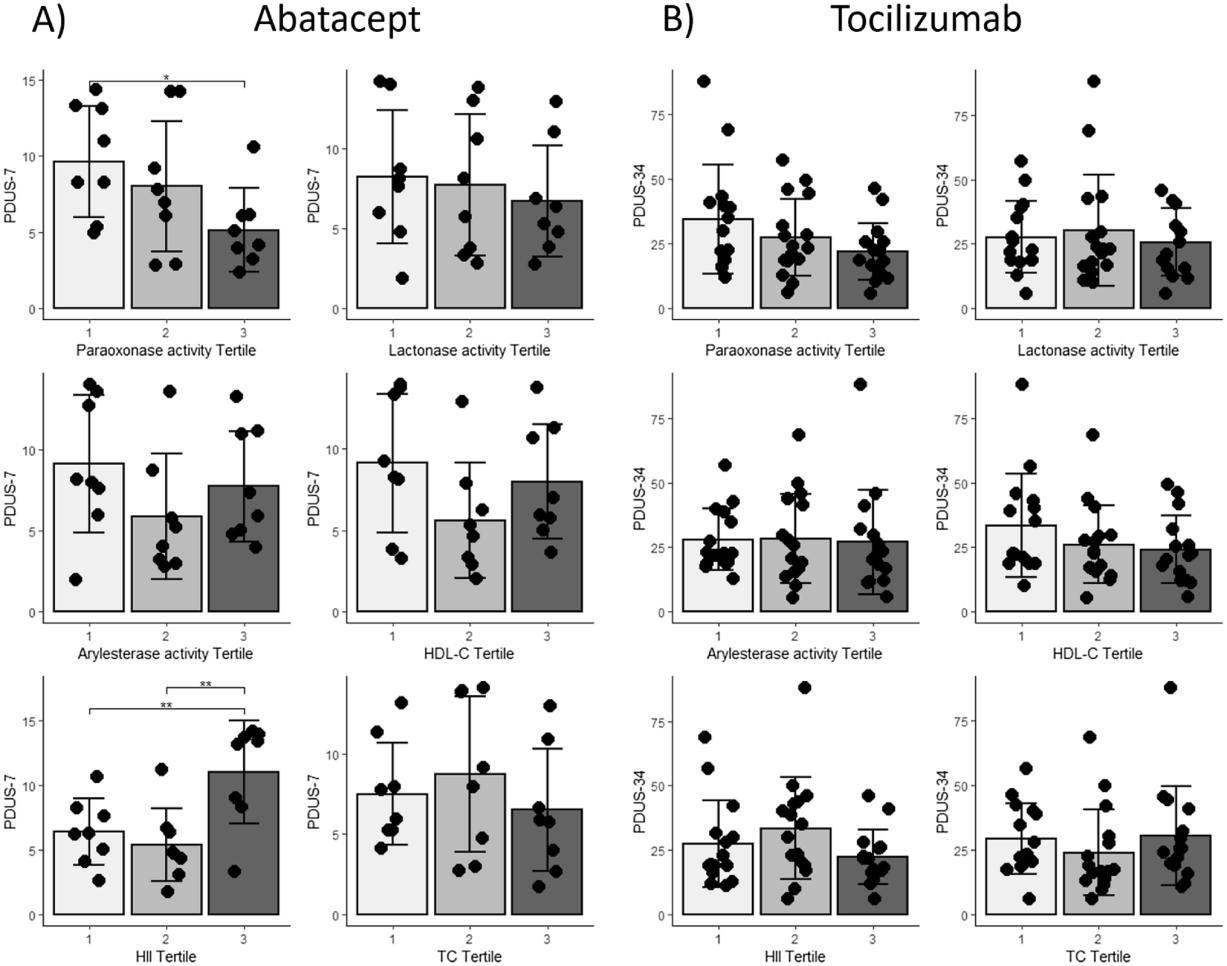
Measures of PDUS grouped by HDL function, PON1 activity and cholesterol tertiles. Panel **A** shows PDUS-7 values in patients participating in the abatacept study grouped by paraoxonase, lactonase, and arylesterase, HII, HDL-C, and TC tertiles. Panel **B** shows PDUS-34 values in patients participating in the tocilizumab study grouped by paraoxonase, lactonase, and arylesterase, HII, HDL-C, and TC tertiles. *p < 0.05 **p < 0.01 ***p < 0.001; pairwise comparisons conducted when ANOVA is statistically significant

Similar relationships between PDUS-7, HDL function, and PON1 activity were noted when examining PDUS-7 in tertiles of HII or PON1 activity. Patients with the most suppressed baseline paraoxonase activity of PON1 (tertile 1) had significantly higher PDUS-7 signal compared to patients with the highest paraoxonase activity of PON1 (tertile 3) (Fig. 2A). Similar trends were observed for PON1 activity assessed by arylesterase and lactonase assays. Patients with the worst overall anti-oxidant function of HDL (tertile 3, highest HII) had significantly higher PDUS-7 signal compared to patients with more protective HDL function in tertiles 1 and 2 (Fig. 2A). Clinical characteristics including demographics, RA disease characteristics, and medication use were similar between HII and paraoxonase tertile groups (Tables 1, 2).

**Table 1:** Clinical and laboratory characteristics of RA patients grouped by baseline HDL function (HDL Inflammatory Index (HII)) tertile

Mean (SD) or *N* (%)	Abatacept				Tocilizumab			
	‘HII’ Tertile 1 (0.97 to 1.84) (*n* = 8)	'HII' Tertile 2 (1.86 to 2.55) (*n* = 8)	'HII' Tertile 3 (2.57 to 7.53) (*n* = 8)	*p* value	'HII' Tertile 1 (0.56 to 1.55) (*n* = 15)	'HII' Tertile 2 (1.55 to 2.43) (*n* = 16)	'HII' Tertile 3 (2.50 to 8.32) (*n* = 15)	*p* value
Age, years	46.5 (15.06)	46.5 (11.72)	56.8 (12.08)	0.13	56.7 (15.51)	50.4 (13.40)	52.9 (15.66)	0.50
Female	8 (100.0%)	8 (100.0%)	6 (75.0%)	0.11	14 (93.3%)	14 (87.5%)	13 (86.7%)	0.81
Hispanic/Latino	3 (37.5%)	1 (12.5%)	1 (12.5%)	0.36	2 (13.3%)	3 (18.8%)	4 (26.7%)	0.65
BMI	22.66 (3.00)	29.64 (8.28)	24.92 (6.70)	0.11	31.23 (6.84)	31.61 (10.11)	27.81 (7.43)	0.39
Disease duration, years	4.0 (4.3)	3.0 (6.6)	6.6 (13.5)	0.47	9.3 (10.49)	11.3 (10.21)	9.1 (8.30)	0.79
Seropositive	6 (75.0%)	6 (75.0%)	5 (62.5%)	0.82	13 (86.7%)	14 (87.5%)	12 (80.0%)	0.82
MTX	5 (62.5%)	2 (25.0%)	4 (50.0%)	0.31	5 (33.3%)	7 (43.8%)	6 (40.0%)	0.84
Current csDMARDs	7 (87.5%)	6 (75.0%)	7 (87.5%)	0.74	8 (53%)	11 (69%)	9 (60%)	0.68
Prednisone	3 (37.5%)	1 (12.5%)	1 (12.5%)	0.36	5 (33.3%)	3 (18.8%)	3 (20.0%)	0.58
ASA	1 (12.5%)	1 (12.5%)	1 (12.5%)	1.00	0 (0.0%)	2 (12.5%)	3 (20.0%)	0.21
Statin	0 (0.0%)	1 (12.5%)	1 (12.5%)	0.58	1 (6.7%)	3 (18.8%)	1 (6.7%)	0.46
Prior bDMARD/tsDMARD	0 (0.0%)	0 (0.0%)	0 (0.0%)	–	11 (73.3%)	12 (75.0%)	15 (100.0%)	0.10
Phys global	6.9 (1.46)	5.5 (1.20)	5.9 (0.99)	0.09	6.6 (1.80)	6.6 (1.50)	5.3 (1.40)	0.045
Pt global	6.5 (1.31)	5.1 (2.80)	6.6 (2.77)	0.40	6.9 (2.46)	7.9 (1.44)	6.8 (2.08)	0.24
SJC28	13.8 (3.96)	12.1 (2.75)	10.4 (2.97)	0.14	13.9 (5.28)	13.1 (5.85)	10.0 (3.16)	0.087
TJC28	12.0 (3.74)	10.8 (4.06)	13.3 (4.20)	0.48	12.5 (5.18)	14.4 (7.78)	13.0 (5.52)	0.69
ESR	49.9(21.03)	46.9(24.39)	32.8(21.32)	0.28	34.0 (19.0, 54.0)	31.5 (19.0, 56.0)	27.0 (15.0, 48.0)	0.83
DAS28	6.59 (0.40)	6.13 (0.76)	6.09 (0.87)	0.31	6.35(0.94)	6.50(1.08)	6.13(0.96)	0.59
CDAI	39.9 (5.14)	33.5 (6.76)	36.1 (6.10)	0.13	39.9(10.26)	42.0(14.04)	35.1(9.56)	0.25
PDUS-7	6.4 (2.56)	5.4 (2.77)	11.0 (4.00)	0.02	–	–	–	–
PDUS-34	–	–	–	–	20.0 (16.0, 32.0)	32.5 (20.0, 43.5)	22.0 (16.0, 26.0)	0.20
IFN_g	58.00 (29.00, 95.25)	85.00 (54.25, 126.00)	116.25 (53.50, 158.00)	0.36	–	–	–	–
IL_10	108.75 (75.00, 165.75)	136.25 (121.00, 158.50)	153.50 (139.25, 206.00)	0.26	–	–	–	–
MiP3a	240.25 (56.50, 253.50)	303.00 (164.00, 594.50)	206.75 (121.00, 369.50)	0.48	–	–	–	–
IL_12p70	83.25 (61.50, 123.75)	43.50 (18.25, 110.25)	106.00 (67.25, 154.75)	0.25	–	–	–	–
IL_3	206.00 (89.50, 349.25)	153.00 (118.00, 298.00)	164.75 (99.00, 235.50)	0.76	–	–	–	–
IL_15	129.50 (61.50, 165.00)	126.00 (93.75, 173.25)	136.00 (73.00, 189.00)	0.83	–	–	–	–
IL_17a	38.00 (16.00, 110.75)	59.75 (26.00, 137.50)	86.75 (41.25, 142.75)	0.35	–	–	–	–
IL_6	208.00 (77.00, 305.50)	172.00 (91.50, 528.25)	188.50 (153.25, 263.00)	0.90	–	–	–	–
IL_17e	187.50 (147.25, 299.75)	249.50 (183.00, 907.50)	216.50 (134.75, 670.75)	0.83	–	–	–	–
IL_27	418.25 (244.25, 488.00)	299.50 (162.50, 796.50)	344.25 (208.25, 853.75)	0.78	–	–	–	–
IL_31	188.25 (137.50, 378.00)	199.50 (157.25, 758.25)	188.50 (133.50, 777.00)	0.94	–	–	–	–
TNFa	180.25 (124.00, 259.50)	382.50 (198.00, 469.50)	212.50 (200.00, 336.50)	0.23	–	–	–	–
IL_28	128.50 (92.75, 212.75)	111.00 (79.50, 173.25)	147.25 (80.50, 173.00)	0.96	–	–	–	–

*BMI* body mass index, *Seropositive* positive ACPA and/or RF, *MTX* methotrexate, *csDMARDS* conventional synthetic disease-modifying anti-rheumatic drug (DMARD), *ASA* aspirin, *bDMARD* biologic DMARD, *tsDMARD* targeted synthetic DMARD, *Phys Global* physician global, *Pt Global* patient global, *SJC* swollen joint count, *TJC* tender joint count, *ESR* erythrocyte sedimentation rate, *DAS* disease activity score, *CDAI* clinical disease activity index, *PDUS* power doppler ultrasound

**Table 2:** Clinical and laboratory characteristics of RA patients grouped by baseline paraxonase activity tertile of PON1

Mean (SD) or *N* (%)	Abatacept				Tocilizumab			
	PON1 Tertile 1 (64.29 to 232.21) (*n* = 8)	PON1 Tertile 2 (236.23 to 463.53) (*n* = 8)	PON1 Tertile 3 (529.64 to 1592.18) (*n* = 8)	*p* value	PON1 Tertile 1 (58.46 to 266.27) (*n* = 15)	PON1 Tertile 2 (267.12 to 500.71) (*n* = 16)	PON1 Tertile 3 (506.14 to 980.41) (*n* = 15)	*p* value
Age, years	55.0 (13.2)	47.3 (13.8)	47.5 (13.5)	0.44	53.1 (15.3)	53.6 (15.3)	53.2 (14.7)	1.00
Female	7 (87.5%)	7 (87.5%)	8 (100.0%)	0.58	13 (86.7%)	13 (81.3%)	15 (100.0%)	0.23
Hispanic/latino	2 (25.0%)	0 (0.0%)	3 (37.5%)	0.17	3 (20.0%)	2 (12.5%)	4 (26.7%)	0.61
BMI	25.81 (6.15)	23.11 (4.27)	28.29 (8.95)	0.32	29.78 (8.82)	30.47 (7.61)	30.47 (8.99)	0.97
Disease duration, years	1.3 (1.2)	6.9 (13.5)	5.5 (7.0)	0.66	9.1 (7.81)	12.1 (11.68)	8.3 (8.81)	0.52
Seropositive	5 (62.5%)	6 (75.0%)	6 (75.0%)	0.82	14 (93.3%)	14 (87.5%)	11 (73.3%)	0.29
MTX	4 (50.0%)	2 (25.0%)	5 (62.5%)	0.31	6 (40.0%)	6 (37.5%)	6 (40.0%)	0.99
Current csDMARDs	7 (87.5%)	7 (87.5%)	6 (75.0%)	0.74	11 (73%)	8 (50%)	9 (60%)	0.41
Prednisone	0 (0.0%)	2 (25.0%)	3 (37.5%)	0.17	5 (33.3%)	2 (12.5%)	4 (26.7%)	0.38
ASA	2 (25.0%)	1 (12.5%)	0 (0.0%)	0.32	1 (6.7%)	1 (6.3%)	3 (20.0%)	0.38
Statin	0 (0.0%)	1 (12.5%)	1 (12.5%)	0.58	2 (13.3%)	1 (6.3%)	2 (13.3%)	0.76
Prior bDMARD/tsDMARD	0 (0.0%)	0 (0.0%)	0 (0.0%)	N/A	12 (80.0%)	13 (81.3%)	13 (86.7%)	0.88
Phys global	6.4 (1.69)	6.4 (1.06)	5.5 (1.07)	0.32	6.5 (1.46)	6.8 (1.81)	5.3 (1.40)	0.04
Pt global	6.5 (1.41)	6.3 (1.67)	5.5 (3.66)	0.70	7.6 (1.96)	7.5 (2.03)	6.6 (2.13)	0.34
SJC28	13.9 (3.83)	12.1 (3.14)	10.3 (2.55)	0.10	14.3 (6.20)	12.4 (4.01)	10.3 (4.35)	0.09
TJC28	12.0 (4.11)	12.1 (4.22)	11.9 (4.19)	0.99	14.0 (8.17)	12.6 (4.96)	13.3 (5.49)	0.83
ESR	52.0 (29.8)	45.3 (13.2)	32.3 (19.8)	0.21	38.0 (24.0, 50.0)	40.5 (16.5, 64.5)	26.0 (16.0, 46.0)	0.41
DAS28	6.52 (0.81)	6.47 (0.42)	5.82 (0.70)	0.08	6.50 (1.10)	6.42 (1.05)	6.06 (0.80)	0.45
CDAI	38.8 (6.73)	37.6 (6.16)	33.1 (5.41)	0.18	42.4 (15.34)	39.3 (8.40)	35.5 (9.93)	0.28
PDUS-7	9.6 (3.62)	8.0 (4.28)	5.1 (2.75)	0.06	–	–	–	–
PDUS-34	–	–	–	–	30.0 (19.0, 41.0)	23.5 (18.5, 38.0)	19.0 (14.0, 26.0)	0.22
IFN_g	152.00 (65.75, 208.00)	69.75 (41.75, 112.25)	58.00 (46.00, 98.25)	0.21	–	–	–	–
IL_10	156.00 (123.50, 434.50)	145.75 (121.00, 153.50)	108.75 (61.00, 148.25)	0.20	–	–	–	–
MiP3a	252.50 (42.00, 369.50)	204.00 (121.00, 577.00)	253.50 (154.50, 320.50)	0.88	–	–	–	–
IL_12p70	135.00 (106.00, 398.50)	46.50 (18.25, 97.00)	63.00 (50.00, 83.25)	0.01	–	–	–	–
IL_3	195.00 (103.50, 308.50)	158.00 (100.25, 240.75)	148.25 (103.75, 308.50)	0.90	–	–	–	–
IL_15	154.00 (95.50, 225.50)	125.50 (73.00, 165.75)	110.50 (93.75, 155.50)	0.56	–	–	–	–
IL_17a	129.50 (45.25, 273.75)	43.75 (33.00, 125.25)	38.00 (23.75, 79.00)	0.28	–	–	–	–
IL_6	220.25 (107.00, 660.00)	177.50 (147.75, 231.25)	215.50 (80.50, 528.25)	0.88	–	–	–	–
IL_17e	332.00 (188.00, 1197.25)	185.50 (79.25, 984.50)	191.75 (156.50, 272.50)	0.35	–	–	–	–
IL_27	458.25 (229.25, 1135.25)	208.25 (155.00, 1088.50)	403.00 (246.00, 452.75)	0.61	–	–	–	–
IL_31	300.50 (153.50, 1188.50)	166.50 (102.50, 1129.50)	221.75 (150.75, 281.25)	0.75	–	–	–	–
TNFa	264.50 (153.25, 502.25)	211.00 (200.00, 409.00)	225.25 (157.50, 321.00)	0.76	–	–	–	–
IL_28	164.75 (106.75, 324.25)	101.00 (53.00, 155.50)	128.25 (89.50, 190.50)	0.31	–	–	–	–

*BMI* body mass index, *Seropositive* positive ACPA and/or RF, *MTX* methotrexate, *csDMARDS* conventional synthetic disease-modifying anti-rheumatic drug (DMARD), *ASA*: aspirin, *bDMARD* biologic DMARD, *tsDMARD* targeted synthetic DMARD, *Phys Global* physician global, *Pt Global* patient global, *SJC* swollen joint count, *TJC* tender joint count, *ESR* erythrocyte sedimentation rate, *DAS* disease activity score, *CDAI* clinical disease activity index, *PDUS* power doppler ultrasound

#### Tocilizumab study

Similar relationships between high baseline PDUS-34 and suppression of the paraoxonase activity of PON1 were noted at baseline in patients in the tocilizumab study. Patients with the highest baseline PDUS-34 score (third tertile PDUS) had the most impaired paraoxonase activity of PON1, which was significantly lower compared to the activity in the patients with the lowest PDUS scores (first tertile PDUS) (Fig. 1B). Higher PDUS-34 also correlated with suppression of circulating paraoxonase activity of PON1 (*r* = − 0.32, *p* = 0.03). Similar trends were noted when evaluating PON1 activity by arylesterase and lactonase assays (Fig. 1B) as well as when examining PDUS in paraoxonase tertiles (Fig. 2B). Relationships between the HII and PDUS-34 tertiles were not noted at baseline in the biologic-experienced RA patients participating in the tocilizumab study (Fig. 2B). Clinical characteristics including demographics, RA disease characteristics, and medication use were similar between PDUS, HII, and paraoxonase tertile groups (Supplementary Table 2, Tables 1, 2).

Further assessment of the HDL particles in tocilizumab patients at baseline demonstrated a potential association of PDUS with an altered HDL protein composition. Patients with the highest PDUS-34 scores (tertile 3) had the lowest HDL- associated apoAI (HDL-apoAI) levels and the highest HDL-associated Hp levels (HDL-Hp) (Fig. 1B). These trends were not statistically significant.

### PDUS scores associate more strongly with HDL function in biologic naïve RA patients compared to other disease activity assessments

#### Abatacept study

Other disease activity measures including the physician global assessments, 28 swollen joint counts, CDAI, and ESR showed trends for association with HDL function, i.e. lower disease activity measures associated with lower HII tertiles (better HDL anti-oxidant function) (Table 1). However, unlike the PDUS-7 tertiles, none of these disease activity measures were significantly different from the tertiles of HDL function (Table 1). In addition, correlations between other disease assessments and HDL function (HII) were noted but were of lesser magnitude compared to correlations with PDUS-7 scores (*r* values = 0.01–0.41, *p* values 0.04–0.96 (physician and patient globals, total/swollen 28 joint counts, DAS28, CDAI, ESR) versus *r* = 0.50, *p* = 0.01 (PDUS-7). Patient characteristics including demographics and background medication use were overall similar in HII tertiles (Table 1).

Similarly, while other disease activity measures showed trends for inverse association with PON1 activity in the abatacept study of biologic naïve patients, PDUS-7 scores and swollen joint count assessments were the RA disease activity measures most closely correlated with impaired paraoxonase activity (*r* = − 0.45 and − 0.50, respectively, *p* = 0.03); lower PDUS and lower numbers of swollen joints were associated with higher paraoxonase activity. PDUS-7, 28 swollen joint counts, and DAS28 showed the greatest differences between tertiles of paraoxonase activity (Table 2). Patient characteristics including demographics and background medication use were overall similar in paraoxonase tertiles (Table 2).

A multiplex cytokine/chemokine panel including IFN-gamma, IL-10, MIP3a, IL12p70, IL-13, IL-15, IL-17a, IL-6, IL-17E, IL-27, IL-31, TNF-a, and IL-28a levels was assessed at baseline and following abatacept treatment. Cytokine/chemokine levels were not consistently associated with any RA disease activity measures at baseline or follow-up and did not associate with PDUS tertiles (Supplementary Table 2).

#### Tocilizumab study

As described above, associations between HII and PDUS-34 were not noted at baseline in the biologic-experienced RA patients participating in the tocilizumab study and were not consistent across other disease activity measures (Table 1). However, paraoxonase activity was associated with several disease activity measures including PDUS-34 which had the highest correlation with paraoxonase activity (*r* = − 0.32 *p* = 0.03) out of the disease activity measures assessed (*r* values = − 0.04 to − 0.31, *p* values 0.03–0.79 (physician and patient globals, tender/swollen 28 joint counts, DAS28, CDAI, ESR). Patient characteristics including demographics and background medication use were overall similar in HII and PON1 tertiles (Tables 1, 2).

### Higher PDUS scores associated with lower HDL and total cholesterol levels

#### Abatacept and tocilizumab studies

Patients with the lowest baseline PDUS scores (tertile 1) had the numerically highest HDL and total cholesterol levels in both the abatacept and tocilizumab studies (Fig. 1). In general, associations of PDUS tertiles with HDL-C levels were stronger than PDUS associations with total cholesterol levels. In the tocilizumab study, the first PDUS tertile with the lowest PDUS signal had significantly higher HDL-C levels compared to the second tertile with a higher PDUS signal (Fig. 1B).

### Improved PDUS scores after treatment with abatacept or tocilizumab associated with improvement in HDL function and increases in cholesterol levels

#### Abatacept

Following treatment with abatacept for a minimum of 3 months, patients with the greatest decreases in PDUS showed modest associations for greater improvement in HDL function, paraoxonase activity, and HDL-C levels compared to patients with the lesser change in PDUS (Table 3). Specifically, large decreases in PDUS (tertile 3 of response) were associated with the largest decreases in the HDL inflammatory index, increases in paraoxonase activity, and increases in HDL-C levels (Table 3).

**Table 3:** Change in HDL inflammatory index, paraoxonase activity, and HDL cholesterol by tertiles of change in power doppler ultrasound signal after treatment with abatacept

	PDUS-7 ∆ Tertile 1 (*n* = 8)	PDUS-7 ∆ Tertile 2 (*n* = 9)	PDUS-7 ∆ Tertile 3 (*n* = 7)
Absolute change in PDUS 7 score	0.63 ± 1.9*	− 2.9 ± 1.1*	− 8.3 ± 2.4
Absolute change in HII	− 0.53 ± 0.85	− 0.22 ± 1.44	− 1.36 ± 2.94
Absolute change in PON1 activity (paroxonase)	− 171 ± 354	− 126 ± 392	30 ± 137
Absolute change in HDL-C	7 ± 25	3 ± 44	25 ± 19

Tertile size variability due to grouping of patients with the same PDUS ∆ score in the same tertile for unbiased comparisons

*PDUS* power doppler ultrasound, *HDL* high-density lipoprotein, *PON1* paraoxonase 1

*p < 0.05 compared to Tertile 3

#### Tocilizumab

**Fig. 3:**
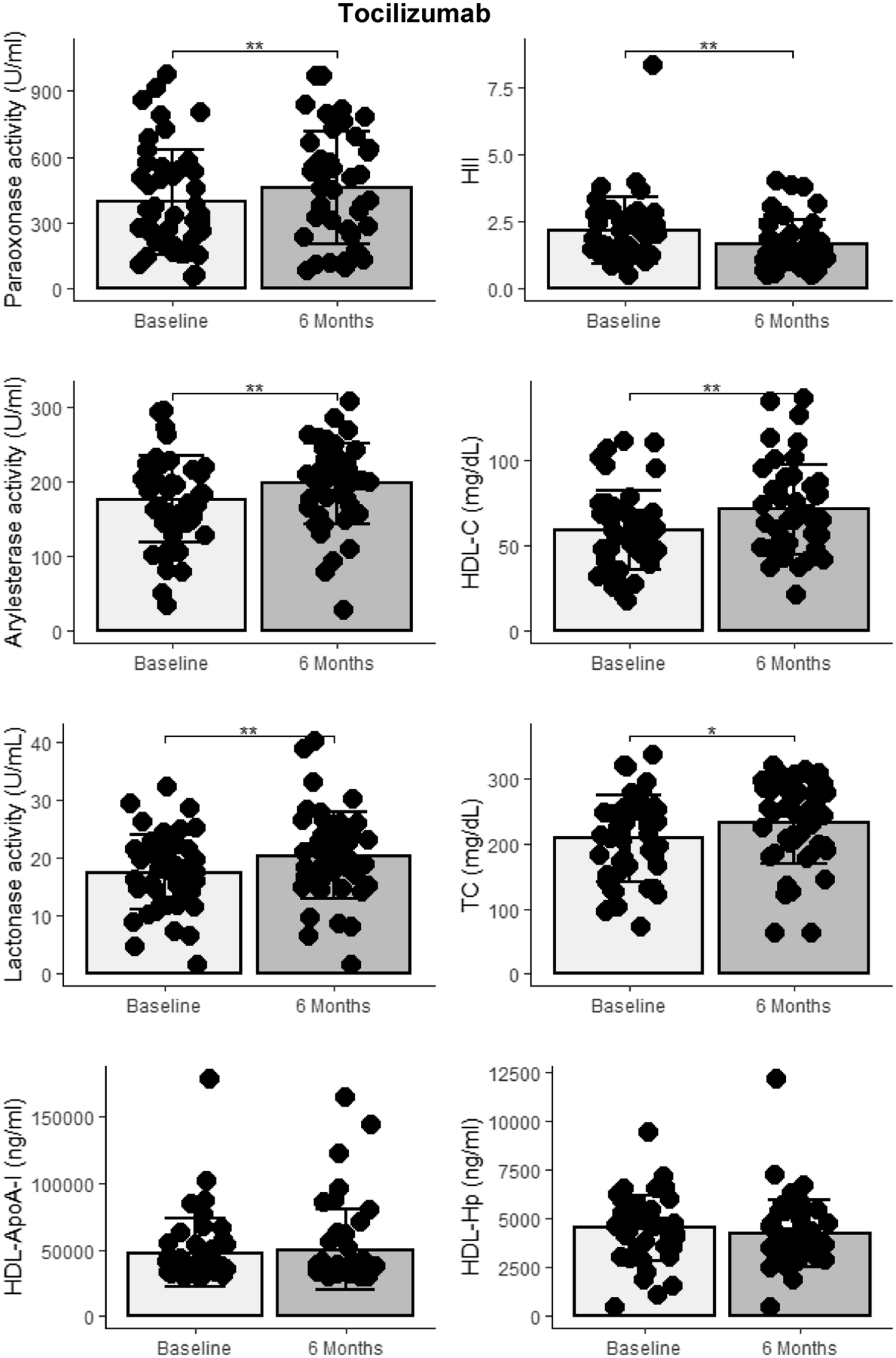
Baseline and 6-month tocilizumab treatment values of HDL function, PON1 activity, and cholesterol. *p < 0.05 **p < 0.01 ***p < 0.001

Treatment with tocilizumab for 6 months was associated with increases in traditional cholesterol levels including significant increases in both total HDL-C and improvements in HDL’s overall antioxidant function (HII), increases in PON1 activity, and non-significant trends for increases in HDL-apoA-I and decreases in HDL-Hp (Fig. 3). Greater decreases in PDUS-34 scores over 6 months were significantly correlated with greater improvements in the HDL particle profile including increases in HDL-C (*r* = − 0.33, *p* = 0.03), HDL-apoA-I (*r* = − 0.31, *p* = 0.03), HDL’s anti-oxidant capacity (HII) (*r* = 0.35, *p* = 0.03).

Associations between other disease assessments (DAS28 and CDAI) and HDL function/structure were noted but were generally of lesser magnitude and consistency across the HDL profile (data not shown). Increases in total and HDL-C levels with tocilizumab treatment correlated with improvement in HDL function (*r* = − 0.41, *p* = 0.005, *r* = − 0.60, *p* < 0.0001, associations of HII with TC and HDL-C, respectively) and PON1 activity (*r* = 0.73, *p* < 0.0001, *r* = 0.43, *p* = 0.003, associations of paraoxonase activity with TC and HDL-C, respectively). Greater increases in cholesterol levels with tocilizumab treatment are associated with greater improvement in HDL’s antioxidant capacity (lower HII) and greater increases in PON1 activity.

## Discussion

Patients with RA have a marked increase in cardiovascular (CV) risk, which has been attributed to both traditional CV risk factors as well as the effects of systemic inflammation, which may accelerate atherosclerosis [1, 4]. The work of our group and others has shown that the HDL particle, which is normally anti-atherogenic in its ability to prevent oxidation of LDL and promote cholesterol efflux from artery wall cells, becomes altered in structure and function in the setting of active systemic inflammation [12, 28]. HDL dysfunction has been linked to CV events in the general population [10], and we recently demonstrated an association of impaired HDL function measured by suppressed PON1 activity with the risk of CV events in a large RA developmental program of the RA therapeutic, tofacitinib [18].

RA-associated inflammation causes increased synovial permeability, allowing HDL to freely pass into the synovial fluid, and our prior work suggests that HDL may be modified and functionally impaired by the pro-inflammatory leucocyte enzyme myeloperoxidase (MPO), and accumulation of pro-inflammatory oxylipins such as 15-Hydroxyeicosatetraenoic acid (15 HETE) in the RA joint [11]. In the current work, we hypothesized that synovial inflammation measured by power Doppler ultrasound would more accurately identify dysfunctional HDL in RA patients than traditional RA disease activity measures and markers of systemic inflammation.

Using two independent clinical trials of abatcept and tocilizumab, we demonstrated that patients with the greatest amount of synovial inflammation at baseline measured by the highest PDUS signal had the greatest impairment in HDL function measured by suppression of PON1 activity. This data was statistically significant in both clinical trials. PON1 is a serum enzyme primarily synthesized in the liver and secreted into the plasma where it associates with HDL [29]. PON1 has multiple anti-inflammatory, anti-atherogenic functions including the metabolism of pro-inflammatory, oxidized lipids in LDL and HDL [9, 30] Low PON1 activity over time is associated with increased risk of CV events in a large RA population participating in the tofacitinib developmental program, after accounting for traditional CV risk factors and lipid profiles [18].

In patients naïve to biological therapy who participated in the abatacept study, the baseline PDUS signal was also associated significantly with the global antioxidant capacity of HDL measured by the HDL inflammatory index, an assessment of the ability of patient HDL to inhibit oxidation of a standard LDL. Patients with the highest amount of synovitis measured by PDUS signal had the worst overall anti-oxidant function of HDL, and patients with the worst HDL function (highest HII) had the highest amount of synovitis measured by PDUS. In contrast, none of the other disease activity measures such as CDAI, DAS28, swollen or tender joint counts, or patient/physician global assessments were significantly different between the tertiles of HDL function. In addition, systemic inflammation measured by ESR was not significantly different between tertiles of HDL function in this work.

The majority of patients in the tocilizumab study had prior biologic or janus kinase inhibitor exposure and similar associations with HII were not noted at baseline as with the biologic naïve patients in the abatacept trial. Our work has previously shown marked associations of disease activity with HDL function and traditional cholesterol levels in another biologic naïve population treated in the TEAR trial [25], and it is plausible that biological exposure played a role in these results. However, patients treated in the tociluzmab trial showed marked improvements in both PON1 activity as well as HDL’s antioxidant function measured by the HII following 6 months of treatment. Trends for other favorable HDL protein particle changes were also noted with modest increases in HDL-apoA-I and decreases in HDL-Hp after 6 months of tocilizumab. ApoA-I makes up approximately 70% of the HDL protein cargo and is the primary protein involved in the promotion of cholesterol efflux from artery walls [31]. Accumulation of haptoglobin in HDL has been associated with impairment in HDL function through inhibition of the activity of the enzyme lecithin:cholesterol acyltransferase (LCAT)**,** which plays a major role in the reverse cholesterol transport [32, 33]. Finally, improvements in the HDL function assessments with tocilizumab therapy were most strongly and consistently associated with changes in PDUS as compared to other disease measures.

Work by Polinski et al. has recently linked inflammatory lipids, in particular the oxylipin 5-HETE, and potentially 15-HETE to the development of future incident inflammatory arthritis in an anticitrullinated protein antibody-positive population, irrespective of inflammatory cytokines [34]. Our group has shown that higher circulating PON1 activity generated by overexpression of the PON1 transgene in an animal model of RA reduces inflammatory arthritis, associating with decreases in 5-HETE and 15 HETE without significant changes in serum cytokine or chemokines [24]. In the current work, measures of HDL function including PON1 activity similarly associated with synovitis by PDUS but did not associate with cytokines or chemokines in the abatacept study. Taken together, this data suggests that further investigation of the inflammatory lipid metabolism in RA as it relates to disease pathogenesis is warranted.

There are limitations to the current work. These were small, open-label clinical trials of different biologic therapies with different durations of therapy, and the biomarkers analyses performed were exploratory. Additional future work of interest includes a larger study evaluating csDMARD use with a single protocol as well as the evaluation of HDL function in patients with clinical remission who have persistently high PDUS. Two different PDUS scoring systems were used for the current trials and, therefore, the results cannot be directly compared*.* However, despite the use of different ultrasound machines, different biologics, different RA medication prior exposure (biologic-naïve vs biologic-exposed), and different numbers of joints scanned by ultrasound (7 vs 34), we found very similar associations of higher levels of synovitis measured by PDUS to HDL dysfunction and suppression of HDL cholesterol levels. This data suggests potentially a more general underlying association of synovitis with HDL function in RA rather than a specific association with one drug treatment pathway. As a smaller point, we do note that while more joints were assessed in the tocilizumab study, the manner of US scoring and obtaining images of the joints were the same.

Additionally, besides systemic inflammation and RA disease activity, there are many factors that can affect HDL function, such as diabetes mellitus, chronic kidney disease, physical activity, and smoking [35–38]. Medications such as glucocorticoids and statins may also affect HDL cholesterol levels and function [23, 39]. The lack of data on several of these baseline risk factors for patients in the trials is a limitation of the current work. Medications such as prednisone and statins, however, remained stable throughout the study period during which cholesterol levels and HDL function were assessed.

Finally, ongoing work is greatly needed to identify the best approach to CV risk assessment in patients with RA. The lipid paradox, recently validated by Giles et al., identified a paradoxical association of suppressed LDL cholesterol levels with increased rather than decreased CV risk in some RA patients, further complicating the clinical CV risk assessment [40]. In the current work, we identified an association of suppressed cholesterol levels with active RA identified by high PDUS scores in both studies. Increases in cholesterol levels with tocilizumab treatment strongly correlated with improvement in HDL function both by the HII and PON1 activity assessments.

In summary, ongoing, active RA over time has been associated with a higher risk of cardiovascular events [4–6]. The current data suggest a mechanism by which active joint inflammation leads to impairment of HDL function including suppressed PON1 activity, and increased CV risk. Ultrasound assessment of synovitis has been included in the ACR/EULAR Classification Criteria for RA to confirm clinical disease findings [41] and EULAR has suggested to use ultrasound when in doubt of the presence of synovitis or when doubting the clinical composite measures in RA patients with concomitant comorbidities like osteoarthritis, fibromyalgia, and obesity [42]. We propose that further work is warranted to confirm these findings in additional larger cohorts as well as to evaluate HDL function in patients with clinical remission who have persistently high PDUS.

## Supplementary Information

Below is the link to the electronic supplementary material.

Supplementary file1 (DOCX 16 KB)

Supplementary file2 (DOCX 18 KB)

## Data Availability

The authors are committed to responsible data sharing. This includes access to anonymized, data sets, as well as other information (eg, protocols or analysis plans). These clinical trial data can be requested by any qualified researchers who engage in rigorous, independent scientific research, and will be provided following review and approval of a research proposal and Statistical Analysis Plan (SAP) and execution of a Data Sharing Agreement (DSA). Data requests can be submitted at any time after the primary manuscript has been published. The data will be accessible for 12 months, with possible extensions considered. For more information on the process, or to submit a request, please contact Dr. Veena Ranganath at vranganath@mednet.ucla.edu.
