# Supplementary material for: Power doppler ultrasound signal predicts abnormal HDL function in patients with rheumatoid arthritis
Source: Rheumatol Int. 2023 Feb 24;43(6):1041–53. doi: 10.1007/s00296-023-05285-7 (PMC10125943; doi:10.1007/s00296-023-05285-7)
Supplement: Supplementary file 1 — Supplementary file1 (DOCX 16 KB) [file 296_2023_5285_MOESM1_ESM.docx]

**Supplementary Table 1.** Descriptive Summary of Demographics

| **Mean(SD) or N(%)** | **Abatacept**  **(n=24)** | **Tocilizumab**  **(n=46)** | **P Value** |
| --- | --- | --- | --- |
| Age, years | 49.9(13.40) | 53.3(14.77) | 0.35 |
| Female | 22(91.7%) | 41(89.1%) | 0.73 |
| Race/Ethnicity | - | - | 0.64 |
| Asian | 3(12.5%) | 3(6.5%) | - |
| Black/African American | 5(20.8%) | 8(17.4%) | - |
| Caucasian/White | 11(45.8%) | 22(47.8%) | - |
| Hispanic/Latino | 5(20.8%) | 9(19.6%) | - |
| Other | 0(0.0%) | 4(8.4%) | - |
| BMI | 25.74(6.79) | 30.25(8.29) | 0.03 |
| Disease duration, years | 4.5(8.75) | 9.9(9.56) | 0.0007 |
| Seropositive | 17(70.8%) | 39(84.8%) | 0.17 |
| ASA | 3(12.5%) | 5(10.9%) | 1.00 |
| Statin | 2(8.3%) | 5(10.9%) | 1.00 |
| MTX | 11(45.8%) | 18(39.1%) | 0.59 |
| Prednisone | 5(20.8%) | 11(23.9%) | 0.77 |
| Prior bDMARD/tsDMARD | 0(0.0%) | 38(82.6%) | <0.0001 |
| Current csDMARDs | 20(83.3%) | 28(60.9%) | 0.05 |

BMI: Body Mass Index, Seropositive: positive ACPA and/or RF, MTX: Methotrexate, csDMARDS: Conventional Synthetic Disease Modifying Anti-Rheumatic Drug (DMARD), ASA: Aspirin, bDMARD: Biologic DMARD, tsDMARD: Targeted Synthetic DMARD

Note: P Values obtained using Student's t-test for continuous variables (except Wilcoxon Rank-Sum for disease duration), and Chi-square (or Fisher's exact test where appropriate) for categorical variables.
