# Supplementary material for: Power doppler ultrasound signal predicts abnormal HDL function in patients with rheumatoid arthritis
Source: Rheumatol Int. 2023 Feb 24;43(6):1041–53. doi: 10.1007/s00296-023-05285-7 (PMC10125943; doi:10.1007/s00296-023-05285-7)
Supplement: Supplementary file 2 — Supplementary file2 (DOCX 18 KB) [file 296_2023_5285_MOESM2_ESM.docx]

**Supplementary Table 2**. Clinical and laboratory characteristics of RA patients grouped by baseline PDUS activity tertile

| **Mean(SD) or N(%)** | **Abatacept** | | | | **Tocilizumab** | | | |
| --- | --- | --- | --- | --- | --- | --- | --- | --- |
|  | **PDUS-7 Tertile 1**  **(2 to 5)**  **(n=9)** | **PDUS-7 Tertile 2**  **(6 to 8)**  **(n=7)** | **PDUS-7**  **Tertile 3**  **(9 to 14)**  **(n=8)** | **P Value** | **PDUS-34 Tertile 1**  **(6 to 18)**  **(n=14)** | **PDUS-34**  **Tertile 2**  **(19 to 28)**  **(n=16)** | **PDUS-34 Tertile 3**  **(30 to 88)**  **(n=16)** | **P Value** |
| Age, years | 53.2(9.80) | 38.7(13.26) | 56.0(12.17) | 0.02 | 51.4(16.09) | 56.1(13.71) | 52.1(15.13) | 0.65 |
| Female | 9(100.0%) | 7(100.0%) | 6(75.0%) | 0.11 | 13(92.9%) | 14(87.5%) | 14(87.5%) | 0.87 |
| Hispanic/Latino | 2(22.2%) | 2(28.6%) | 1(12.5%) | 0.74 | 3(21.4%) | 1(6.3%) | 5(31.3%) | 0.20 |
| BMI | 29.49(7.46) | 21.05(2.15) | 25.63(6.62) | 0.04 | 30.18(7.62) | 33.74(8.79) | 26.82(7.30) | 0.06 |
| Disease duration, years | 5.0(6.6) | 3.1(3.9) | 5.3(13.7) | 0.38 | 8.5(10.26) | 9.8(8.84) | 11.3(10.04) | 0.73 |
| Seropositive | 6(66.7%) | 5(71.4%) | 6(75.0%) | 0.93 | 10(71.4%) | 15(93.8%) | 14(87.5%) | 0.22 |
| MTX | 2(22.2%) | 4(57.1%) | 5(62.5%) | 0.19 | 9(64.3%) | 6(37.5%) | 3(18.8%) | 0.04 |
| Current csDMARDs | 5(55.6%) | 7(100.0%) | 8(100.0%) | 0.02 | 11(78.6%) | 10(62.5%) | 7(43.8%) | 0.15 |
| Prednisone | 2(22.2%) | 2(28.6%) | 1(12.5%) | 0.74 | 1(7.1%) | 4(25.0%) | 6(37.5%) | 0.15 |
| ASA | 1(11.1%) | 1(14.3%) | 1(12.5%) | 0.98 | 3(21.4%) | 1(6.3%) | 1(6.3%) | 0.31 |
| Statin | 1(11.1%) | 0(0.0%) | 1(12.5%) | 0.63 | 1(7.1%) | 1(6.3%) | 3(18.8%) | 0.45 |
| Prior bDMARD/  tsDMARD | 0(0.0%) | 0(0.0%) | 0(0.0%) | - | 12(85.7%) | 12(75.0%) | 14(87.5%) | 0.61 |
| Phys Global | 5.4(1.01) | 6.1(1.07) | 6.8(1.58) | 0.12 | 4.9(0.83) | 6.3(1.82) | 7.2(1.33) | 0.0003 |
| Pt Global | 4.8(3.11) | 7.0(1.63) | 6.8(1.39) | 0.11 | 6.6(1.91) | 7.3(2.30) | 7.7(1.89) | 0.38 |
| SJC28 | 10.4(1.88) | 12.1(3.85) | 13.9(3.83) | 0.12 | 10.3(3.79) | 11.6(4.76) | 14.9(5.60) | 0.03 |
| TJC28 | 10.6(3.40) | 13.4(4.35) | 12.4(4.24) | 0.35 | 12.2(5.15) | 11.8(5.40) | 15.8(7.31) | 0.13 |
| ESR | 39.3(13.9) | 33.7(17.2) | 55.8(30.3) | 0.14 | 28.5 (15.0, 48.0) | 28.5 (19.0, 55.5) | 40.0 (21.5, 57.0) | 0.63 |
| DAS28 | 5.90(0.44) | 6.30(0.79) | 6.70(0.73) | 0.07 | 6.02(0.84) | 6.22(1.0) | 6.70(1.0) | 0.15 |
| CDAI | 31.2(4.2) | 38.7(4.99) | 40.5(5.63) | 0.002 | 34.1(8.15) | 36.9(11.10) | 45.6(12.33) | 0.014 |
| PDUS-7 | 3.8(1.09) | 7.0(1.00) | 12.4(1.85) | - | - | - | - | - |
| PDUS-34 | - | - | - | - | 13.5 (11.0, 17.0) | 22.5 (19.5, 25.0) | 42.5 (37.0, 48.0) | - |
| IFN_g | 70.00 (53.00, 116.00) | 55.00 (37.00, 69.50) | 122.50 (101.50, 158.00) | 0.10 | - | - | - | - |
| IL_10 | 99.50 (69.00, 142.00) | 118.00 (112.00, 251.00) | 154.00 (149.75, 187.25) | 0.12 | - | - | - | - |
| MiP3a | 293.00 (99.00, 328.00) | 69.00 (15.00, 249.00) | 253.75 (204.75, 369.50) | 0.09 | - | - | - | - |
| IL_12p70 | 60.00 (56.00, 68.00) | 81.50 (21.00, 119.50) | 124.50 (85.25, 170.50) | 0.09 | - | - | - | - |
| IL_3 | 128.50 (79.00, 249.00) | 138.00 (100.00, 325.00) | 205.25 (131.25, 281.00) | 0.72 | - | - | - | - |
| IL_15 | 93.50 (41.00, 117.00) | 155.00 (105.00, 176.50) | 146.50 (97.50, 177.00) | 0.19 | - | - | - | - |
| IL_17a | 40.00 (21.50, 74.50) | 49.50 (26.00, 138.00) | 116.50 (63.00, 142.75) | 0.18 | - | - | - | - |
| IL_6 | 194.50 (69.50, 541.00) | 258.00 (157.50, 300.50) | 153.25 (107.00, 225.25) | 0.81 | - | - | - | - |
| IL_17e | 187.00 (160.50, 306.50) | 192.50 (93.50, 211.50) | 408.25 (207.00, 1197.25) | 0.29 | - | - | - | - |
| IL_27 | 219.50 (189.00, 421.50) | 284.00 (204.50, 548.00) | 458.25 (306.00, 1135.25) | 0.47 | - | - | - | - |
| IL_31 | 160.50 (141.00, 223.50) | 171.00 (134.00, 457.00) | 364.50 (180.00, 1188.50) | 0.35 | - | - | - | - |
| TNFa | 357.00 (208.50, 400.00) | 152.00 (119.00, 210.00) | 227.50 (208.50, 397.00) | 0.07 | - | - | - | - |
| IL_28 | 81.00 (70.50, 122.50) | 124.00 (90.50, 216.00) | 173.00 (147.25, 192.25) | 0.18 | - | - | - | - |

BMI: Body Mass Index, Seropositive: positive ACPA and/or RF, MTX: Methotrexate, csDMARDS: Conventional Synthetic Disease Modifying Anti-Rheumatic Drug (DMARD), ASA: Aspirin, bDMARD: Biologic DMARD, tsDMARD: Targeted Synthetic DMARD, PDUS: Power Doppler Ultrasound
